# Network depth affects inference of gene sets from bacterial transcriptomes using denoising autoencoders

**DOI:** 10.1093/bioadv/vbae066

**Published:** 2024-05-08

**Authors:** Willow Kion-Crosby, Lars Barquist

**Affiliations:** Helmholtz Institute for RNA-based Infection Research (HIRI)/Helmholtz Centre for Infection Research (HZI), 97080 Würzburg, Germany; Faculty of Medicine, University of Würzburg, 97080 Würzburg, Germany; Helmholtz Institute for RNA-based Infection Research (HIRI)/Helmholtz Centre for Infection Research (HZI), 97080 Würzburg, Germany; Faculty of Medicine, University of Würzburg, 97080 Würzburg, Germany; Department of Biology, University of Toronto, Mississauga, ON L5L 1C6, Canada

## Abstract

**Summary:**

The increasing number of publicly available bacterial gene expression data sets provides an unprecedented resource for the study of gene regulation in diverse conditions, but emphasizes the need for self-supervised methods for the automated generation of new hypotheses. One approach for inferring coordinated regulation from bacterial expression data is through neural networks known as denoising autoencoders (DAEs) which encode large datasets in a reduced bottleneck layer. We have generalized this application of DAEs to include deep networks and explore the effects of network architecture on gene set inference using deep learning. We developed a DAE-based pipeline to extract gene sets from transcriptomic data in *Escherichia coli*, validate our method by comparing inferred gene sets with known pathways, and have used this pipeline to explore how the choice of network architecture impacts gene set recovery. We find that increasing network depth leads the DAEs to explain gene expression in terms of fewer, more concisely defined gene sets, and that adjusting the width results in a tradeoff between generalizability and biological inference. Finally, leveraging our understanding of the impact of DAE architecture, we apply our pipeline to an independent uropathogenic *E.coli* dataset to identify genes uniquely induced during human colonization.

**Availability and implementation:**

https://github.com/BarquistLab/DAE_architecture_exploration.

## 1 Introduction

Gene expression regulation is vital for bacteria to survive environmental changes. Minor gene expression shifts can trigger nutrient uptake systems (Schalk and Guillon 2013, Davies *et al.* 2021), flagella assembly for mobility (Armitage and Berry 2020), biofilm formation (de la Fuente-Núñez *et al.* 2013), and other responses necessary for survival of harsh conditions or antibiotic treatment (Seo *et al.* 2015, Gollan *et al.* 2019). Specific transcriptomic programs are linked to host colonization and pathogenicity as well (Fang *et al.* 2016). Transcriptomics offers a comprehensive view of gene expression, enabling exploration of fundamental questions about bacterial behavior, infection mechanisms, persistence, and resistance. Inference of microbial gene regulatory networks has a rich history. Early efforts applied diverse methodologies (Marbach *et al*. 2012), including regression-based approaches (Bonneau *et al.* 2006, 2007), mutual information techniques (Faith *et al.* 2007), and correlation methods (Langfelder and Horvath 2008), to extensive microarray datasets. The advent of bulk RNA sequencing has revitalized the field, driving development of new unsupervised techniques to determine regulatory networks in bacteria (Sastry *et al.* 2019, Tan *et al.* 2020, Yuan *et al.* 2022).

In recent years, deep learning has emerged as a new tool for transcriptomic analysis (Zou *et al.* 2019). Deep learning's strengths lie in the inclusion of hidden layers between input and output, creating network depth that enables intricate, hierarchical data processing, yielding cutting-edge performance across various applications (LeCun *et al.* 2015, Eldan and Shamir 2016). Autoencoders (AEs), a neural network variant, are trained by iteratively compressing each datapoint into a lower-dimensional representation through a bottleneck (BN) layer before attempting to reconstruct the original datapoint (Hinton and Salakhutdinov 2006). Optimal training is achieved by minimizing the difference between the original and reconstructed data, ensuring that the low-dimensional compression is meaningful. Denoising autoencoders (DAEs) introduce random noise to the input, leading to more robust recovery of feature relationships (Vincent *et al.* 2008).

DAEs have been applied to the unsupervised inference of co-expressing sets of genes from microarray data. DAEs have been shown to outperform other methods for gene set inference such as ICA (Tan *et al.* 2016, 2017), a highly competitive tool according to independent benchmarks (Saelens *et al.* 2018). In Tan *et al.* (2016, 2017), the authors used shallow DAEs, or networks where the input layer is directly connected to the BN layer. This choice allowed for direct model interpretability through examination of the weights connected to each BN node. Additionally, the authors developed an ensemble method (Tan *et al.* 2017) where many networks are trained to circumvent model instability, as networks trained from different initial conditions yield different results when trained on limited data sets. However, DAEs offer immense flexibility and the operator can decide on layer count, layer widths, and notably, BN width. The impact of these architectural choices on downstream analysis remains unclear.

**Figure 1.**
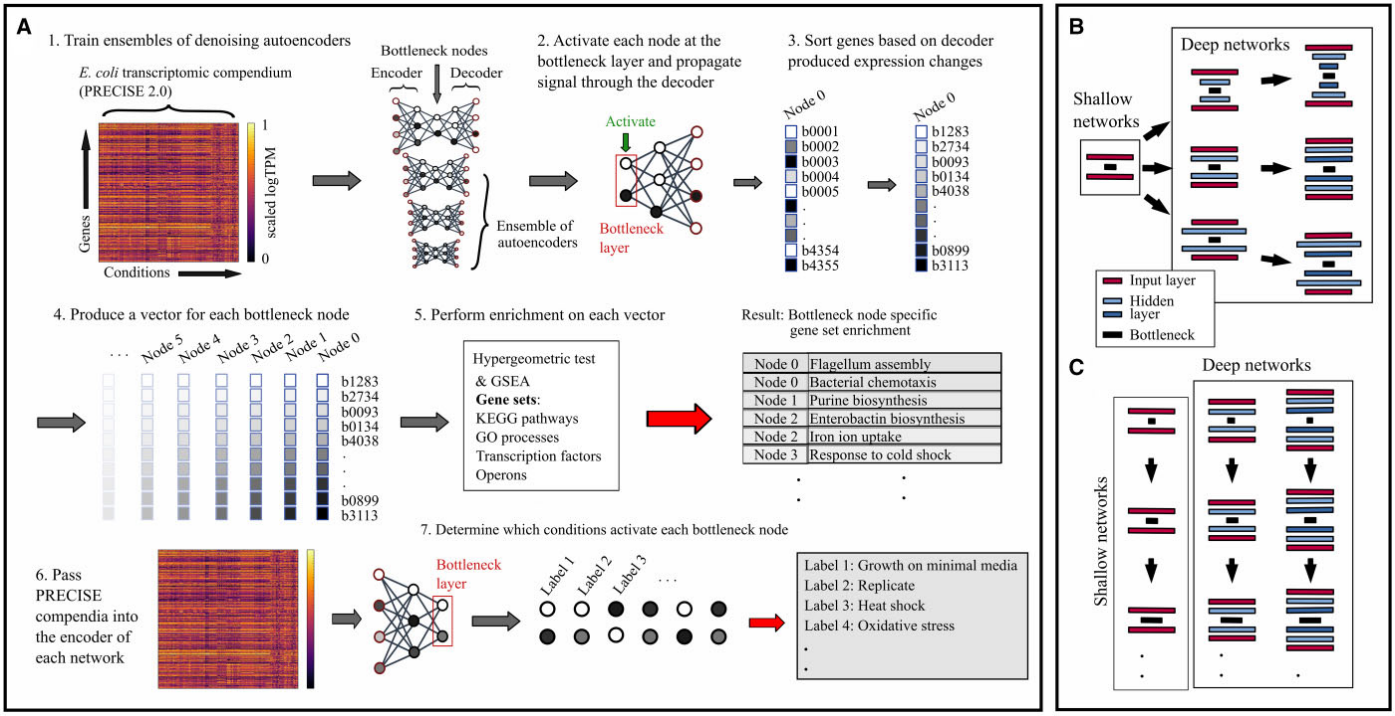
DAE architecture exploration pipeline. (A.1) Each ensemble is trained on the PRECISE 2.0 compendium. (A.2) Each bottleneck node is activated, (A.3) and the activation is propagated through the decoder. The resulting decoder outputs are then sorted. (A.4) This process is done for each bottleneck node. (A.5) Gene set enrichment analysis is then performed on these vectors based on KEGG pathways, operons, regulators, and GO processes. (A.6) Finally, PRECISE 2.0 is passed through the encoder. (A.7) Each of the nodes, and their corresponding gene sets, are then associated with each growth condition. (B) The first set of seven architectures we have explored. All architectures have a bottleneck of 50 nodes and an input/output matching the number of genes. (C) The set of variable–bottleneck networks.

Here, we have devised a gene set inference pipeline for bacterial expression data using DAEs (Fig. 1), accommodating both shallow and deep networks by examining changes in predicted gene expression when activating each BN node separately. Throughout this manuscript, we refer to both the DAE-predicted sets of coregulated genes, and the sets of genes based on known curation, as “gene sets.” We have employed this pipeline to assess how the choice of DAE architecture effects gene set recovery by training on the PRECISE 2.0 RNA-seq compendium (Lamoureux *et al.* 2021) for *Escherichia coli*. Finally, we have leveraged our understanding of DAE architecture to identify new gene sets uniquely linked to human infections in a uropathogenic *E.coli* (UPEC) transcriptomic dataset (Subashchandrabose *et al.* 2014). This work extends the usage of DAEs to deep models and illustrates the advantages of network depth in inferring coherent gene sets from transcriptomic data.

## 2 Methods

### 2.1 Construction of the DAEs with various architectures

**Figure 2.**
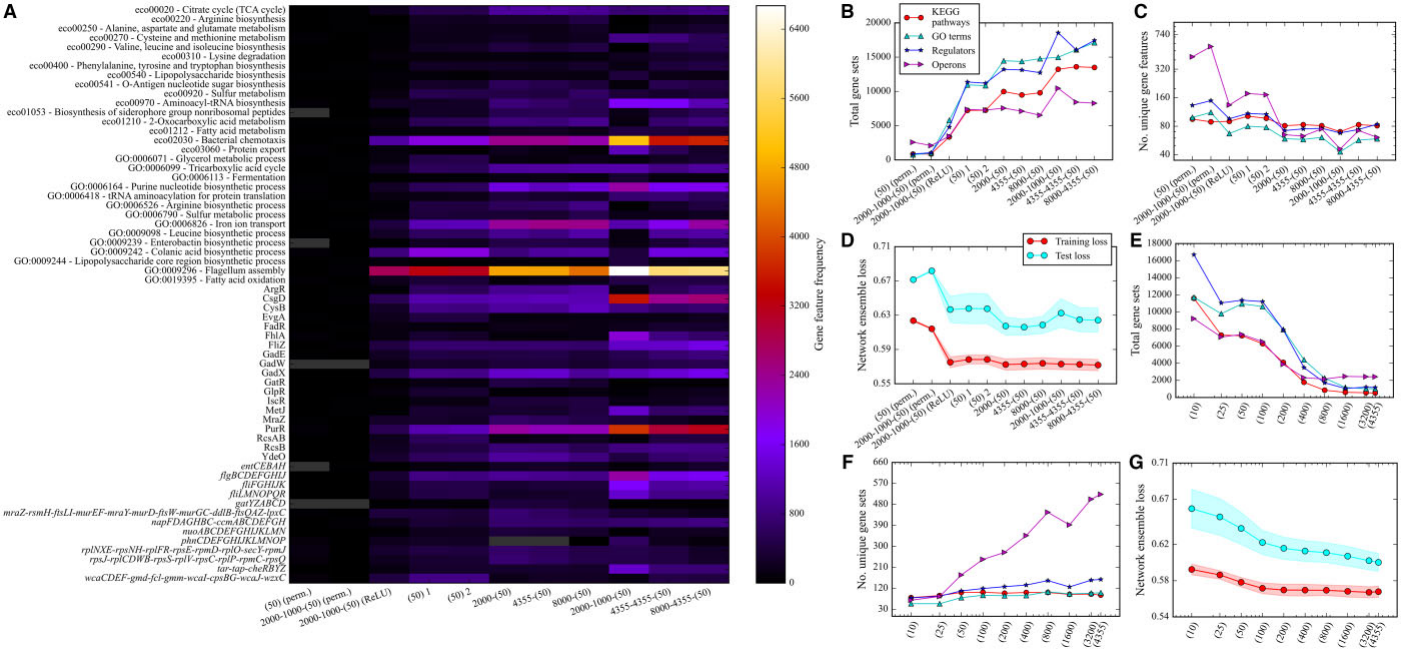
Enrichment for each network architecture. (A) The frequency of recovered gene sets for each architecture. Network architecture notation along the *x*-axis is written as *N_1_*—*N_2_*—…—(*N_B_*) where *N_x_* and *N_B_* are the number of nodes in layer *x* and the bottleneck, respectively, also illustrated in Fig. 1B. Cell color represents gene set frequency; grey denotes no occurrence. Only gene sets which were recovered from at least 3% of nodes for at least a single architecture are shown. The first two columns show the results of shallow and deep ensembles trained on permuted data, followed by the deep network parameterized by ReLU activation functions trained on unpermuted data. The next columns show repeats of the shallow ensemble. The labels on the *y*-axis represent recovered KEGG pathways, GO processes, regulators, and operons, respectively. (B) The total number of recovered gene sets, including redundancies, for architectures in Fig. 1B. (C) The number of unique gene sets. (D) The losses for ensembles of 100 networks with single standard deviations shown. (E) The total number of gene sets for the full ensemble of shallow DAEs for various bottlenecks. (F) The number of uniquely recovered gene sets. (G) The losses for ensembles of 100 networks for each shallow network compression with single standard deviations shown as envelopes.

AEs were implemented using the Python package Keras (Chollet and Others, 2015). Models typically used sigmoid activation functions at every layer including the BN and output layers. For the 2000–1000–(50) ReLU network (Fig. 2), all layers excluding the BN and output were parameterize with ReLU functions. The weights of each layer for all networks were randomly initialized based on the Glorot distribution, and bias vectors initialized with zeros. The weight matrices which make up the decoder of each network are tied, i.e. they are the transpose of the corresponding weight matrices of each encoder.

### 2.2 Adjustment of BN layer size

When adjusting the BN layer of each network architecture, the total number of networks in each ensemble is changed to keep the BN nodes constant. For instance, when reducing the number of nodes in the BN from 50 to 25, the number of networks in the ensemble was increased to 200, such that there are still 5000 nodes. For comparison, the case where the number of networks is held constant and the number of BN nodes is allowed to vary was examined, by dividing the results shown in Fig. 2E and F by the number of networks in each ensemble (see Supplementary Fig. S7).

### 2.3 AE network training and cross-validation

All networks were trained using the Adam optimization algorithm. Data corruption was employed during training such that 10% of entries of each input RNA-seq experiment is randomly set to zero during each training step.

Early stopping was employed to improve generalizability. To do so, each data set was randomly partitioned into an 80% training and a 10% validation set. An example of training and validation trajectories for a single architecture is shown in Supplementary Fig. S8. A 10% test set was separately partitioned to determine a final score for each model. Before training, logTPM values were scaled between 0 and 1 for the training, validation, and test sets separately. Local search was performed over all training parameters including the learning rate and batch size using the validation score as a metric with batch shuffling enabled.

### 2.4 Collection of various gene sets

Regulon and operon information was retrieved from RegulonDB (Santos-Zavaleta *et al.* 2019) and KEGG pathways using the KEGG Python module from the bioservices package, in October of 2021. GO processes were extracted from the RefSeq annotation of the complete genome of *E.coli* str. K-12 substr. MG1655 with accession number NC_000913.

### 2.5 Statistical tests to associate BN nodes with activation or suppression of specific gene sets

A hypergeometric test was employed to determine gene set enrichment for each BN node vector, including a Benjamini–Hochberg correction for multiple hypothesis testing. Additionally, since many such vectors are examined for representative gene sets (5000 for an ensemble of 100 networks each with 50 BN nodes), a Bonferroni correction was applied such that the expected number of false discoveries is <1 for a full network ensemble. To achieve this, we set a threshold of 10^−8^ on the false discovery rate. This number is based on both the number of BN nodes and number of KEGG pathways (110), GO processes (110), regulons (408), and operons (816) used for enrichment.

Gene set enrichment analysis (GSEA) (Subramanian *et al.* 2005) was performed using each full BN vector as a preranked list, and again controlled for false discoveries. For both statistical tests, we limit the gene set size to at most 40 genes to avoid performing statistical tests on broad categories.

### 2.6 Determination of conditions associated with specific gene sets, and selection of consensus experimental conditions

To identify conditions associated with up-regulation of specific gene sets, the compendium was passed into all 100 encoders of each network ensemble. The conditions which activate BN nodes associated with the gene set of interest were then examined. To determine a consensus experimental condition, after performing this previous procedure for all three network architectures, (50), 2000–(50), and 2000–1000–(50), a single condition was randomly selected which appears in the overlap of the three resulting sets of top 50 conditions.

### 2.7 Comparison of pairwise distribution statistical tests

**Figure 3.**
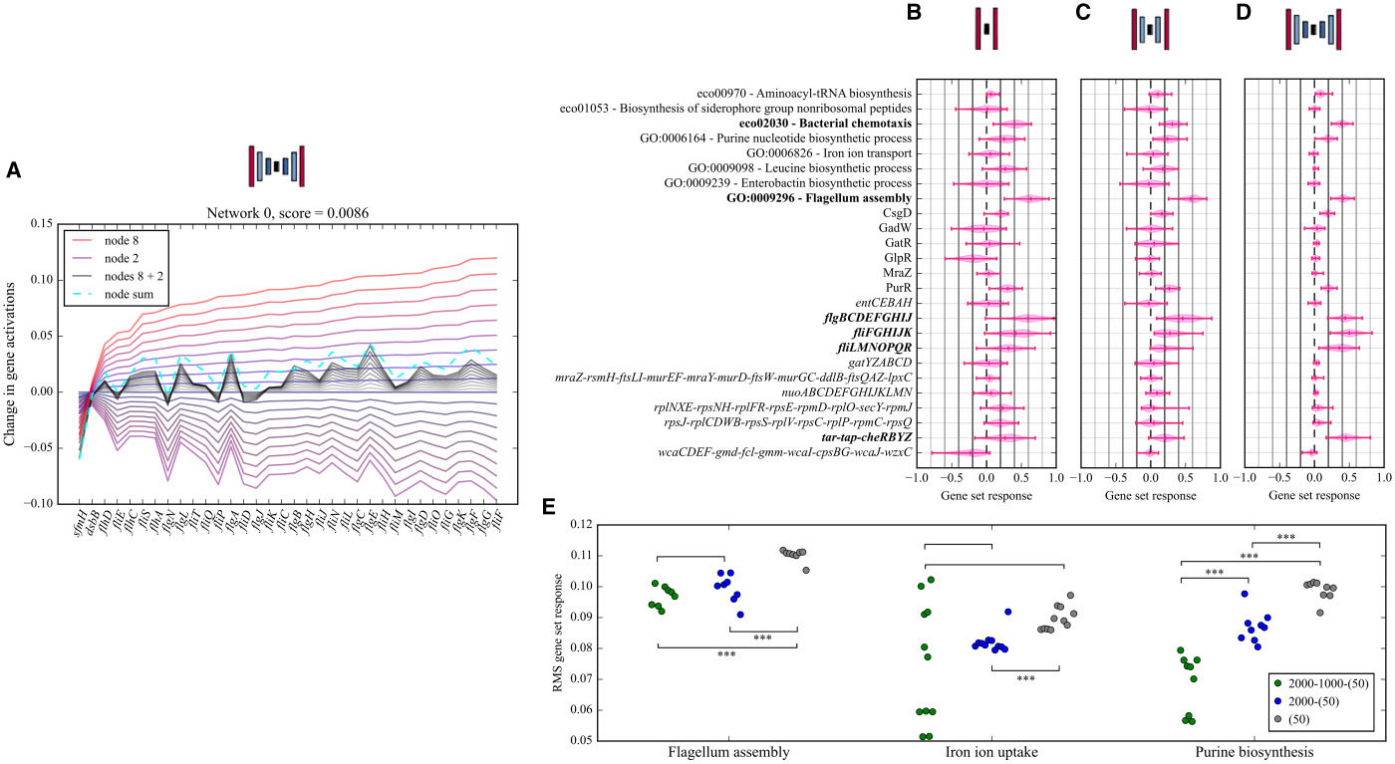
DAE-gene set response to individual experimental conditions. (A) The decoder output for the flagellum assembly GO process gene set when a single activating node from a 2000–1000–(50) network (node 8) is activated gradually from 0 to 1 (red lines of increasing intensity). The purple curves below the *x*-axis represent a single suppressing node (node 2), and the grey curves correspond to both nodes being activated simultaneously. The dashed cyan line is the sum of the maximum decoder responses for both nodes 8 and 2, and the score at the top of the figure is the L1-norm between the final grey curve and the cyan line. (B–D) Gene set responses for the most impacted gene sets when a single randomly-selected experiment which activates flagellum assembly is passed through the encoders of the full ensembles. (E) RMS response values across all gene sets and networks for the three architectures shown in B–D, computed for sets of eight experimental conditions which all three network architectures agree upregulate the indicated pathway. Pairwise comparisons between distributions performed using a Wilcoxon rank sum test (see Section 2): (***) indicates a *P* value of <.001, while no * indicates a *P* value >.05.

To perform comparisons between the enrichments of all pairs of network architectures shown in Fig. 2A, distributions of the number of enriched gene sets per network were first generated. Wilcoxon rank sum tests were then performed between these distributions to test if the means were significantly different. When testing between architectures of the same depth, two-sided tests were performed, while single-sided tests were performed between architectures of different depths. Single-sided Wilcoxon rank sum test were also performed for the distributions of gene set responses shown in Fig. 3E.

### 2.8 Identification of UTI-specific nodes and gene sets

To identify UTI-specific nodes in the PRECISE 2.0-trained 2000–1000–(50) ensembles, we passed each of the HUTI, UR, and LB experiments individually into the encoder of each network. Nodes which have a value of >0.5 for all HUTI, and <0.5 for all of the other two conditions, were considered UTI-specific. These nodes were then activated individually, and this activation was propogated through the decoder of each network, following the standard procedure of our pipeline, to produce a UTI-specific gene set.

### 2.9 Gene and protein data acquisition

**Figure 4.**
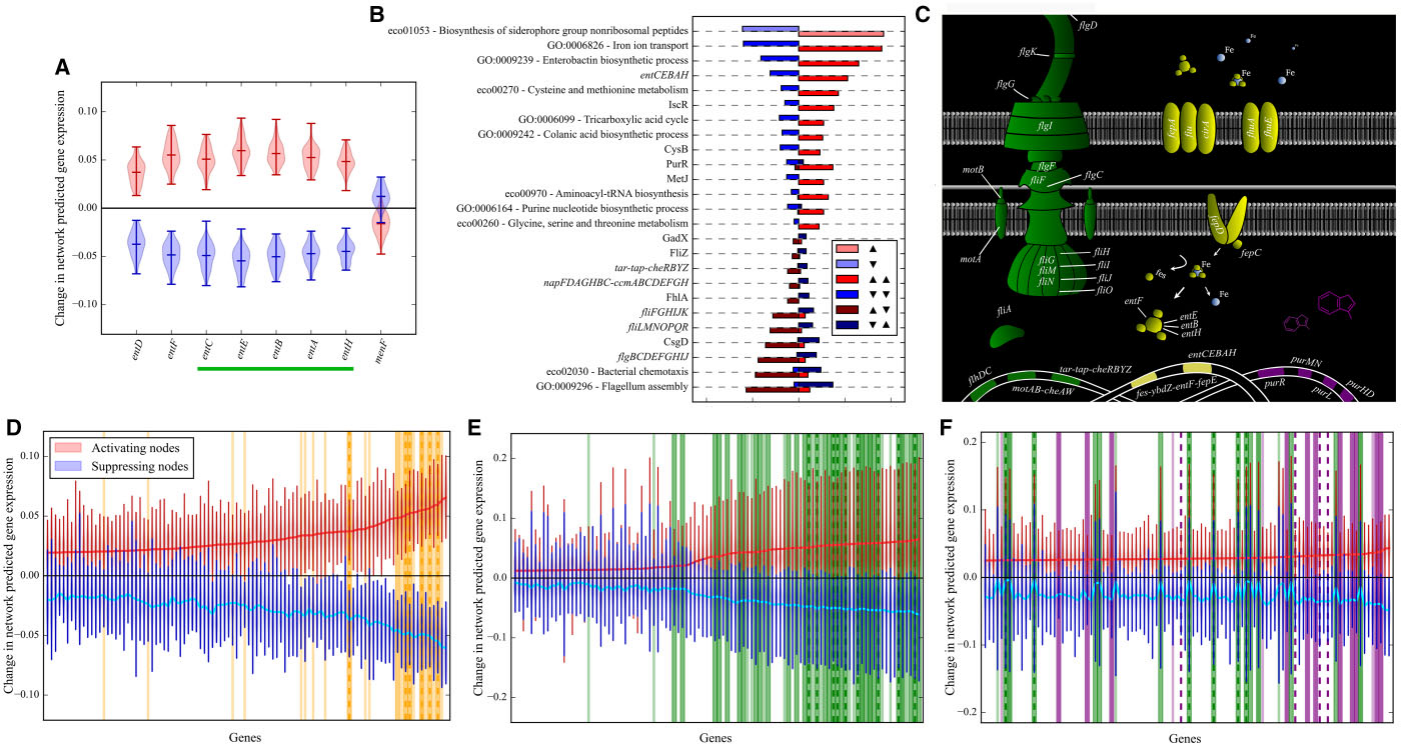
Predicted connection between gene sets by the 2000–1000–(50) network. (A) Distributions of DAE-predicted expression changes for genes in the siderophore biosynthesis KEGG pathway with red and blue corresponding to activating and suppressing nodes, respectively. The green bar indicates cooperonic genes. (B) The number of gene sets which are also associated with siderophore biosynthesis nodes. Whether this siderophore biosynthesis pathway is activated (upward arrow) or suppressed (downward arrow) is indicated by the left set of arrows in the legend. The number of siderophore biosynthesis nodes which either activate (light red) or suppress (light blue) this pathway are shown as the first set of bars. Only gene sets which occur in at least 20% of siderophore biosynthesis nodes are shown. Left-facing bars indicate the number of nodes which suppress (downward arrow) the corresponding gene set on the *y*-axis, while right-facing bars indicate activation (upward arrow). (C) A simplification of three processes recovered from PRECISE 2.0 and a rough reconstruction of the locations and functions of the majority of the proteins involved in the gene sets highlighted in D–F. Colors match categories in D–F. (D) The top 100 most-activated genes, and their corresponding predicted expression changes (red for activating nodes and blue for suppressing nodes), when siderophore biosynthesis nodes are activated. The dark yellow bands indicate genes in the siderophore biosynthesis KEGG pathway, while the light-thin bands and vertical-dashed lines indicate genes in the GO processes for iron ion transport and enterobactin biosynthesis, respectively. (E) and (F) show the same quantities, but for nodes associated with the flagellum assembly, and purine biosynthesis. The dark-green bands, light-green, thin bands, and dashed green lines indicate genes in the flagellum assembly GO process, bacterial chemotaxis KEGG pathway, and the *flgBCDEFGHIJ* operon, respectively. Finally, the dark-purple bands, light-purple, thin bands, and dashed purple lines indicate genes in the purine biosynthesis GO process, the PurR regulon, and the aminoacyl-tRNA biosynthesis KEGG pathway. These gene sets were chosen because they represent the most-frequent co-associations (see Supplementary Figs S6B and S7B).

The locations and functions of the various proteins in Fig. 4C are taken from Chang and Liu (2019), Fujii *et al.* (2017), Johnson *et al.* (2021), and Liu and Ochman (2007) for the genes relating to the flagellum assembly GO process, and Neumann *et al.* (2018), Zhang *et al.* (2020), Stintzi *et al.* (2000), and Kanehisa *et al.* (2023) for genes involved in iron uptake. We take additional supporting information from Santos-Zavaleta *et al.* (2019).

### 2.10 Validation on permuted data

To generate the permuted version of the PRECISE 2.0 compendium, gene expression values for each gene were independently shuffled such that the distributions of expression values per gene are preserved, but the gene–gene correlations and condition specificity are destroyed.

## 3 Results

### 3.1 A Pipeline for AE architecture exploration

To explore how network architecture impacts biological inference, we trained 100 DAEs with a variety of architectures. For training we used the PRECISE 2.0 *E.coli* K-12 RNA-seq compendium (Lamoureux *et al.* 2021), consisting of 815 experiments (Fig. 1A.1). After training, we activate each node at the BN and propagate this signal through the decoder. This provides a prediction of gene expression which we examine to determine the biological processes these nodes represent through GSEA (Fig. 1A.1–5). When a known gene set is found to be enriched in this expression vector, we refer to this as the gene set being “recovered” from the node.

### 3.2 Increased network depth amplifies biological inference

We explored three basic network architectures using our pipeline (Fig. 1B**;** see also Section 2). Here, we use the notation:


$$N_{1}-N_{2}-\ldots -(N_{B}),$$

where *N_x_* and *N_B_* are the number of nodes in layer *x* and the BN, respectively. Encoder arrangements mirror that of the decoder. Our first architecture implements gradual compression, drawing from early AE work (Hinton and Salakhutdinov 2006), a widely-adopted choice in the AE literature (Srivastava *et al.* 2014, Eraslan *et al.* 2019, Lotfollahi *et al.* 2021). We also included architectures without compression until the BN, and architectures which first expand by ∼2-fold. We performed our pipeline for ensembles of 100 networks for each of three architectures and two network depths, along with two ensembles for a shallow network architecture to illustrate pipeline stability.

Shallow and deep networks are clearly different, with deeper networks enriched for more known gene sets (Fig. 2A and B): All pairwise comparisons of networks of different depths result in *P* values <10^−9^ from a Wilcoxon rank sum test (see Section 2 and Supplementary Table S1). Differences between architectures of equal depth are typically more nuanced with few comparisons resulting in *P*-values <.05 by a Wilcoxon rank sum test (Supplementary Table S1). This is evident in Fig. 2B where the networks 2000–(50), 4355–(50), and 8000–(50) recover comparable KEGG pathways, GO processes, and regulators. Shallow networks recover the most unique gene sets (Fig. 2C), though infrequently. These results resemble permuted data, suggesting that recovered unique gene sets are a poor measure of network quality. We also observe that the choice of activation function impacts gene set inference, with ReLU activiation functions leading to recovery of fewer gene sets (Fig. 2B) although the training and test losses remain unaffected (Fig. 2D). Therefore, deeper networks parameterized with sigmoid activation functions tend to more robustly code biological gene sets with comparable test losses (Fig. 2D).

### 3.3 Balance between biological inference and test loss defines optimal compression

Next we examined the network compression (Fig. 1C), an often challenging choice. In terms of inference power (the total number of enriched gene sets recovered from all BN nodes from each entire ensemble), wider networks perform worse (Fig. 2E) and are sensitive to operons (Fig. 2F). Narrower networks are forced to learn broader pathways, while wider networks code individual operons. There is a plateau in inference power between 25 and 100 nodes (Fig. 2E) with improved test loss (Fig. 2G), suggesting a tradeoff between reconstruction accuracy and inference. We have also performed a local scan over compressions for the deeper architectures and see a similar trend, although the optimum appears to be closer to 25 nodes for 4355-(*N_B_*) networks (see Supplementary Fig. S1).

### 3.4 Deep networks explain the same data with fewer pathways

We next investigated the node activation in each experimental condition (Fig. 1A.6–7). We often observe that a complex combination of nodes activates from single conditions. To quantify this, we observe that the relationship between node activation and decoder response is approximately linear (Fig. 3A). The sum of the decoder outputs when two nodes are independently activated is reasonably approximated by the output when both nodes are activated simultaneously. This approximation appears to be accurate across nodes enriched for three distinct gene sets for networks of various depths (Supplemenary Fig. S2) as quantified by the L1-norm.

Therefore, we define the gene set response, *ɸ*, to each experimental condition as:


$$ɸ\left(x_{i}\right)\equiv \Sigma_{j\epsilon \mathrm{nodeset}}z_{j}\left(x_{i}\right)\Delta y_{j}\left(z_{j}=1\right),$$

where *z_j_*(*x_i_*) is the activation of node *j* when experiment *x_i_* is passed through the encoder, and *Δy_j_*(*z_j_* = 1) is the average log-fold change in predicted gene expression when *z_j_*(*x_i_*) is set to 1 with all other nodes set to 0. This metric represents the average log-fold change in gene expression for a single gene set in response to a single experimental condition.

To investigate how network depth affects *ɸ*, we randomly chose a single RNA-seq experiment that activates flagellum assembly nodes in all three architectures (see Section 2). For all 100 networks and architectures, we computed *ɸ* for every gene set (Fig. 3B–D). We see that deeper networks respond with few gene sets. These include eco02030—bacterial chemotaxis, GO0009296—flagellum assembly, the operons *flgBCDEFGHIJ*, *fliFGHILK*, *fliLMNOPQR*, and *tar-tap-cheRBYZ*: all associated with flagellum assembly or motility. To quantify this selectivity, we have taken the RMS (root mean square) deviation from 0 of the distributions of *ɸ* illustrated in Fig. 3B–D for each architecture, since a lower RMS indicates fewer activated gene sets. An RMS of 0 would indicate no gene set activation, while each gene set activation distribution shifted from zero would contribute to a higher RMS. We performed this calculation for a set of eight consensus experimental conditions which activate three different gene sets (see Section 2). The deeper networks tend to lower the response RMS, although this is not true for several iron ion uptake conditions (see Fig. 3E). This is because many of the iron ion uptake nodes found in the 2000–1000–(50) architecture also down-regulate flagellum assembly (see Fig. 4B) adding diversity to the response.

### 3.5 AE BN nodes define gene sets beyond their associated enriched terms and predict regulatory connections between disparate processes

We observe that nodes are often associated with multiple related gene sets, implying that nodes represent biological processes beyond the associated enriched terms. To investigate this, we used the architecture 2000–1000–(50) that typically produces the most independent pathway activation (see Fig. 3D–E), and showed the greatest inference power (see Fig. 2B), and examined how each of the siderophore biosynthesis nodes changes the expression for only the genes within this gene set (Fig. 4A). Although most of the genes in this set show an expected expression change, *menF* stands out as potentially randomly distributed. The function of *menF* is performed by *entC* during enterobactin biosynthesis (Dahm *et al.* 1998, Buss *et al.*, 2001) suggesting that these nodes represent this latter process.

The other most-frequent gene sets in these nodes suggest that the DAEs are learning the mechanism by which iron is actively transported across the outer membrane of *E.coli* via the ferric-siderophore complex (Stintzi *et al.*, 2000) (see Fig. 4B). This pathway is shown in the middle of Fig. 4C where 15 of the top 20 genes predicted by the DAEs (top 100 shown in Fig. 4D) are explicitly shown. The iron–sulfur cluster regulon for the transcription factor IscR also appears in 38 of the 92 siderophore biosynthesis activating nodes, and 15/61 of the suppressing nodes. This suggests that these nodes also capture iron–sulfur cluster formation. This is further evidenced by the comparable presence of the cysteine and methionine metabolism KEGG pathway (43/92 activating and 19/61 suppressing; Fig. 4B) gene set as cysteine is often the sulfur source in Fe-S cluster biogenesis (Ayala-Castro *et al.* 2008).

We explored which genes show the greatest predicted change in expression when these siderophore biosynthesis nodes are activated to further uncover what these nodes represent biologically. Most genes in panel D of Fig. 4 can be trivially connected to iron uptake or iron–sulfur cluster assembly, as even the genes in the *nrdHIEF* operon (explicitly shown in Supplementary Fig. S3) are regulated by the transcription factors Fur and IscR. To demonstrate that the gene set reported by the DAEs shown in Fig. 4D and Supplementary Fig. S3 is indeed up/downregulated in the top/bottom 15 conditions which activate siderophore biosynthesis nodes (see Section 2), we have computed the *z*-scores from these conditions for these genes and plotted these values alongside the distributions of *z*-scores across the entire compendium (Fig. S3). With the exceptions of *yddA/B*, this result supports the validity of this *de novo* gene set generation method.

We also note that disparate processes are often enriched within the same nodes. For instance, for the 92 nodes associated with *activating* siderophore biosynthesis, 57 of these nodes also *suppress* the flagellum assembly GO process (Fig. 4B). This signal is also recovered in the shallower network 2000–(50), but far less strikingly (Supplementary Fig. S4), further supporting the use of the deeper architectures. We also see the converse relationship in a similar proportion. This implicit relationship between iron uptake and cell motility is not easily explained by the activity of the Fur repressor, which regulates many of the genes in both pathways. This inverse relationship has been observed previously (Inoue *et al.* 2007, Zhang *et al.* 2020), and was proposed to be mediated by expression of the protein YdiV (Zhang *et al.* 2020). However, we find the *ydiV* gene to be actively suppressed by siderophore biosynthesis nodes, and we also observe this downregulation directly in the data (see Supplementary Fig. S3). Purine biosynthesis nodes also often activate flagellum assembly (see Supplementary Fig. S5B), and to a lesser extent the converse is also true (Supplementary Fig. S6B). This pattern is reflected in the top 100 genes for these nodes (Fig. 4F). This mixing is also biased toward activating nodes suggesting that flagellum assembly and purine biosynthesis are only upregulated together. Therefore, node enrichment frequently suggests potential regulatory interactions between processes.

### 3.6 Pretrained AEs reveal gene sets uniquely associated with uropathogenic *E.coli* infection

**Figure 5.**
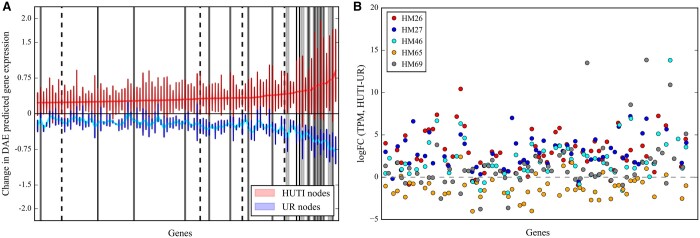
Prediction of the UTI-specific gene set through the PRECISE 2.0-trained 2000–1000–(50) networks. (A) The top 100 most-activated genes (red indicating the 14 HUTI-specific nodes and blue indicating 4 UR-specific nodes). The wide, light-grey bands indicate genes of CSPs, while the darker, thin bands indicate genes of the Qin prophage. The solid black lines represent genes in the Rac prophage, and the dashed lines genes of the DLP12 prophage. (B) logFCs between HUTI and UR for all five strains from the UTI data set for the same genes shown in A. No marker is shown when the gene is missing in the corresponding strain.

In this final section, we have used the previous lessons to discover infection-specific gene sets in the transcriptome of *E.coli* during a urinary tract infection (UTI). To accomplish this, we apply the same approach we previously employed (Schumacher *et al.* 2023; Section 2) to identified gene sets specifically responsive to acid stress in *E.coli*. To identify nodes from the PRECISE 2.0-trained 2000–1000–(50) networks which activate solely during UTI using the transcriptomes of five uropathogenic *E.coli* isolates (HM26, HM27, HM46, HM65, and HM69) grown in lysogeny broth (LB), urine from healthy donors (UR), and directly from participants with urinary tract infections (HUTI) (Subashchandrabose *et al.*, 2014). Out of the 5000 nodes (50 BN nodes from an ensemble of 100 networks), we have identified 14 nodes which only activate in HUTI amongst the three conditions. The gene set defined by the HUTI-specific nodes show a negative change in expression for another group of UR-specific nodes (Fig. 5A), supporting the hypothesis that these genes are UTI-specific compared to either urine or LB *in vitro* growth. Examining the logFC from the original data set between UR and HUTI shows that the majority of genes are indeed upregulated in HUTI (Fig. 5B), with the exception of HM65 indicating some strain-specificity.

We next examined the functions of these HUTI-specific genes. These include six genes encoding cold shock proteins (CSPs), *cspA*, *cspB*, *cspF*, *cspG*, *cspH*, and *cspI*. The *lpxP* gene encoding the cold-inducible lipid A palmitoleoyltransferase was also present. The HUTI samples were stabilized using RNAprotect to avoid liquid nitrogen use (Subashchandrabose *et al.* 2014), making cold shock unlikely. CSPs, particularly CspC and CspE, have been shown to be important for survival in a variety of stress conditions, including infection (Michaux *et al.* 2017, Yair *et al.* 2022). Our results suggest other CSPs may play additional roles in infection.

Many of the genes in our HUTI-specific gene set are encoded on cryptic prophages. Thirteen are part of the Qin prophage, constituting nearly a third of this island. Other genes include three from the Rac prophage, and four from the DLP12 prophage. Prophages such as Qin are known to be “cryptic” as their machinery for excision and virus production have been rendered inert. The genes encoded on these islands appear to be involved in a range of infection-related processes, including stress tolerance, biofilm formation, and antibiotic resistance (Wang *et al.* 2010).

Next, we investigated the hypothesis that induction of these HUTI-specific genes forms part of a general stress response. Twelve of the top 30 RNA-seq experiments from PRECISE 2.0 which active these nodes correspond to treatment with paraquat to induce oxidative stress. Another 12 experiments correspond to antibiotics ciprofloxacin, ceftriaxone, meropenem, or trimethoprim–sulfamethoxazole growth (Tan *et al.* 2020), also suggesting the HUTI-specific gene set is stimulated by a variety of stresses.

## 4 Discussion

Here, we have explored how network architecture impacts inference from bacterial expression data through a pipeline we developed to recover gene sets from ensembles of DAEs. We observed an approximately linear relationship between the activations of the nodes and the predicted expression of each gene, and as a consequence we are able to include and interpret deep networks in our analysis, in contrast to previous work (Tan *et al.* 2016, 2017). We have found that deeper networks produce more concise gene sets and recover them with a greater frequency. We have also found that nodes often recover coordinate regulation of disparate processes. Finally, we demonstrate that deep DAEs can be used to recover gene sets *de novo*, and apply the technique we used in (Schumacher *et al.* 2023) to transcriptomic data from uropathogenic *E.coli* to identify a gene set unique to the infection process consisting of CSPs and prophage genes, potentially involved in response to stressors encountered in the host.

When varying the BN width, we found that the test loss of each network ensemble continues to improve even as the training scores converge (Fig. 2G). While the wider networks could be finding solutions in which the data is passed from encoder to decoder without alteration, leading to near perfect training and test scores, it appears they are biased toward solutions with more biological significance. Wider networks recovered more operons regardless of network depth (see Supplementary Fig. S2B and E and Fig. 2F) and networks with 4355 BN nodes (i.e. no compression) still recover known gene sets in addition to the greatest number of unique operons (Fig. 2E and F). This implicit regularization characteristic of deep learning has been described previously for image classification (Barron 1994; Neyshabur *et al.* 2014).

Because of this ever-improving test loss, we have defined optimal compression as a balance between the improving test loss and the decrease in inference power for wider networks (Fig. 2E). However, this decrease appears to be a consequence of our choice to hold the total number of nodes constant by varying the number of trained networks in each ensemble. The number of inferred gene sets *per node* decreases as we increase the number of nodes. Alternatively, if we allow the number of networks to vary, we observe that the number of recovered gene sets *per network* only increases as we increase the BN layer (see Supplementary Fig. S7). Therefore, with wider networks, individual processes are automatically being assigned to individual nodes similar to the concept of disentanglement in variational autoencoders (VAEs).

Our approach to exploring DAE architectures for gene set inference could be adapted to other types of AEs, such as VAEs. A number of studies have explored architecture choices in VAEs, but only by examination of the test score from held-out data on eukaryotic data sets (Lotfollahi *et al.* 2020, 2023, Lotfollahi *et al.*, 2021, Chow *et al.* 2022). Our linear decomposition of node activations may serve as a complementary alternative to other approaches to AE interpretation, such as training a linear decoder alongside a deep encoder (Svensson *et al.* 2020, Lotfollahi *et al.* 2023).

One advantage of AE analysis is the ability to discover patterns across whole RNA-seq compendia in a hypothesis-free fashion. This not only allows for the study of other less-well-characterized bacteria through our pipeline, given solely RNA-seq data for that organism, but also contrasts with traditional studies of transcriptional regulators, which are often done in defined inducing conditions. The associations we uncovered indicate several instances of process co-regulation, not easily explained by known regulatory mechanisms. For instance, the regulon of the transcription factor PurR is associated with ∼50% of siderophore biosynthesis nodes (Fig. 4B). Another example is the predicted inverse relationship between flagellum assembly and siderophore biosynthesis (Fig. 4B). Thus, our method serves as a tool for generating hypotheses linking expression of biological processes and provides a link to the conditions in which connections may be further explored.

## Supplementary Material

vbae066_Supplementary_Data

## Data Availability

The DAE pipeline, implemented in Python, with examples and scripts for training and analyzing networks with variable architectures is available at the GitHub repository https://github.com/BarquistLab/DAE_architecture_exploration. This repository features a README file with instructions on how to run the analysis, and also includes a tutorial. Additionally, data files with the values shown in, and genes corresponding to, Figs 4D–F and 5A, as well as a table with all gene sets used for enrichment are included in this repository.
